# CCR2 promotes monocyte recruitment and intestinal inflammation in mice lacking the interleukin-10 receptor

**DOI:** 10.1038/s41598-021-04098-7

**Published:** 2022-01-10

**Authors:** Shorouk El Sayed, Izabel Patik, Naresh S. Redhu, Jonathan N. Glickman, Konstantinos Karagiannis, El Sayed Y. El Naenaeey, Gamal A. Elmowalid, Ashraf M. Abd El Wahab, Scott B. Snapper, Bruce H. Horwitz

**Affiliations:** 1 Division of Gastroenterology, Hepatology, and Nutrition, Department of Pediatrics, Boston Children’s Hospital, 300 Longwood Avenue, Boston, MA 02420 USA; 2 Faculty of Veterinary Medicine, Department of Microbiology, Zagazig University, Zagazig, Ash Sharkia Egypt; 3 Present Address: Morphic Therapeutic, Waltham, MA USA; 4 Department of Pathology, Beth Israel Deaconess Medical Center, Boston, MA USA; 5 Center for Biologics Evaluation and Research, US Food and Drug Administration, Silver Spring, MD USA; 6 Division of Gastroenterology, Brigham and Women’s Hospital, Boston, MA USA; 7 Division of Emergency Medicine, Boston Children’s Hospital, Boston, MA USA

**Keywords:** Inflammatory bowel disease, Inflammation, Monocytes and macrophages, Interleukins

## Abstract

Macrophages are a heterogeneous population of mononuclear phagocytes abundantly distributed throughout the intestinal compartments that adapt to microenvironmental specific cues. In adult mice, the majority of intestinal macrophages exhibit a mature phenotype and are derived from blood monocytes. In the steady-state, replenishment of these cells is reduced in the absence of the chemokine receptor CCR2. Within the intestine of mice with colitis, there is a marked increase in the accumulation of immature macrophages that demonstrate an inflammatory phenotype. Here, we asked whether CCR2 is necessary for the development of colitis in mice lacking the receptor for IL10. We compared the development of intestinal inflammation in mice lacking IL10RA or both IL10RA and CCR2. The absence of CCR2 interfered with the accumulation of immature macrophages in IL10R-deficient mice, including a novel population of rounded submucosal Iba1^+^ cells, and reduced the severity of colitis in these mice. In contrast, the absence of CCR2 did not reduce the augmented inflammatory gene expression observed in mature intestinal macrophages isolated from mice lacking IL10RA. These data suggest that both newly recruited CCR2-dependent immature macrophages and CCR2-independent residual mature macrophages contribute to the development of intestinal inflammation observed in IL10R-deficient mice.

## Introduction

Inflammatory bowel disease (IBD) is a complex genetic disease that represents an interplay of environmental factors and genetic susceptibility associated with a dysregulated immune response against gut microenvironmental triggers^1–3^. IBD includes both ulcerative colitis (UC) and Crohn’s disease, which exhibit relapsing and remitting gastrointestinal mucosal inflammation. Genome-wide association studies have determined that polymorphisms in genes associated with bacterial sensing, innate immunity, and Th17 pathways, such as NOD2, STAT3, IL10, and IL23R, are associated with an altered risk of developing IBD^4,5^. In addition, rare homozygous loss-of-function mutations in IL10R are strongly associated with severe and difficult-to-treat intestinal inflammation during infancy^6–8^. These prior studies suggested that understanding the proper regulation of inflammatory pathways by IL10R signaling is essential for developing effective therapies for colitis^9,10^. IL10 is an anti-inflammatory cytokine secreted primarily by myeloid, B and T cells. Its receptor is a heterotetrametric complex that consists of two α subunits (IL10RA) and two β subunits (IL10RB)^11,12^. While the α subunits are unique to IL10R, the β subunits are shared with receptors for other cytokines, including IL22, IL26 and INF-λ^13,14^. Following engagement by IL10, IL10R activates the nonreceptor tyrosine kinases JAK1 and TYK2, inducing downstream signaling pathways, including STAT3 phosphorylation and nuclear translocation^10^. It has been shown that the function of IL10R on innate immune cells and macrophages in particular is essential to prevent colitis^15–17^. Moreover, Bernshtein and colleagues have shown that mice specifically lacking IL10RA in macrophages (*Cx3cr1*^*Cre*^*Il10ra*^*fl/fl*^) develop spontaneous colitis^18^. Macrophages from these engineered mice exhibit a profound inflammatory signature characterized by elevated expression of *Il23a, Il12b*, and *Ccl5*. Pointing toward a critical role for macrophage pro-inflammatory gene expression in the etiology of colitis, mice lacking both *Il10ra* and *Il23a* specifically in macrophages are protected from colitis^18^.

The mature intestinal macrophage pool is constantly replenished through the influx of Ly6C^hi^ blood monocytes that initiates at the time of weaning and is dependent on microbial colonization^19^. Following extravasation, these monocytes proceed through a series of maturation steps, including downregulation of Ly6C expression, acquisition of MHCII and increasing expression of CX3CR1^20^. Recently, it has been demonstrated that two distinct mononuclear phagocyte cells defined as CD11c^+^CD206^int^CD121b^+^ and CD11c^−^CD206^hi^CD169^+^ originate from common CCR2^+^ precursors during maturation of colon MPs. These two populations have distinct gene expression profiles and anatomic localization. CD11c^+^CD206^int^CD121b^+^ macrophages localize close to the tips of the villi and express genes associated with immune effector function, while CD11c^−^CD206^hi^CD169^+^ macrophages are localized below the base of the crypt and within the submucosa and express genes related to cell recruitment and tissue regeneration^21^. These results suggest that monocytes recruited into the colon can develop distinct phenotypes and functions.

It has previously been shown that under homeostatic conditions, the accumulation of monocyte-derived macrophages in the colon depends on CCR2^19,22^. Bain and colleagues observed that mice lacking CCR2 had a reduced number of Ly6C^+^ colonic macrophages compared to age-matched wild-type mice, suggesting that CCR2 was necessary for monocyte recruitment into the colon. In addition, irradiated mice reconstituted with equal mixtures of wild-type and CCR2-deficient bone marrow cells demonstrated elevated ratios of wild-type to CCR2-deficient macrophages within the colon, indicating that CCR2 is essential for the accumulation of monocyte-derived macrophages. However, this result may not solely be the result of a defect in the recruitment of CCR2-deficient monocytes into the intestine, as there was a considerable reduction in the ratio of CCR2-deficient to wild-type monocytes in circulation^19^. In fact, this is consistent with prior results suggesting that CCR2 signaling is necessary for monocyte mobilization from the bone marrow^23,24^.

While blood monocytes recruited into the colon of healthy mice quickly differentiate into macrophages with a mature phenotype, this maturation process appears disrupted during inflammation. Within the colon of mice treated with DSS, there is a marked increase in the total number of colonic macrophages as well as an increase in the proportion of Ly6C^+^ macrophages relative to mature Ly6C^−^ macrophages^20^. Interestingly, compared to wild-type mice, CCR2-deficient mice treated with DSS exhibited a reduction in the percentage and number of F4/80^+^TLR2^+^ macrophages in the colon. These authors demonstrated that these F4/80^+^TLR2^+^ cells corresponded to a CX3CR1^int^Ly6C^hi^CCR2^+^ population observed in the colon of CX3CR1^Gfp^ mice challenged with DSS, suggesting that the absence of CCR2 inhibited the recruitment of Ly6C^+^ macrophages. Furthermore, following adoptive transfer of equal mixtures of wild-type and CCR2-deficient bone marrow cells into host mice, there was a considerable defect in the accumulation of intestinal macrophages derived from the CCR2-deficient bone marrow compared to intestinal macrophages derived from wild-type bone marrow. These results suggest that the presence of CCR2 is essential for the accumulation of Ly6C^+^ inflammatory macrophages observed within the colon of DSS-treated mice^25^.

In contrast to the studies described above for DSS colitis, the severity of colitis in IL10-deficient mice was not reduced by the deletion of CCR2, and differences in the frequency of the predominant macrophage populations within the colon of these doubly deficient mice were not detected. However, the fractions of colonic macrophages that expressed both Ly6C^+^ and MHCII^+^ cells were not explicitly evaluated in this study^26^. Therefore, it remains an open question whether CCR2 is in fact necessary for the accumulation of Ly6C^+^MHCII^+^ monocyte-derived macrophages observed within the colon of mice lacking IL-10R signaling.

We have shown that IL10R-deficient mice (C57BL/6) carrying the cytokine deficiency-induced colitis susceptibility locus (*Cdcs1*) develop spontaneous colitis within 3–4 weeks of birth. Colitis in these mice is characterized by a large increase in the total number of colonic macrophages and an increase in the proportion of Ly6C^+^MHCII^+^ monocyte-derived macrophages, similar to that observed in DSS-treated mice^17^. Furthermore, we and others have demonstrated that macrophages play an essential role in the development of disease pathogenesis^10,15,17,18^. However, it remains unclear whether the accumulation of Ly6C^+^MHCII^+^ monocyte-derived macrophages is necessary for the development of disease or whether this accumulation depends on CCR2. Here, we demonstrate that CCR2 is essential for the accumulation of Ly6C^+^MHCII^+^ macrophages within the colon of IL10R-deficient mice and that colitis was significantly less severe in these mice. Thus, we suggest that the CCR2-dependent accumulation of Ly6C^+^MHCII^+^ macrophages in the colon of IL10R-deficient mice exacerbates the development of intestinal inflammation.

## Results

### Colitis is less severe in *Cdcs1*^+*/*+^*Il10ra*^−*/*−^ mice lacking *Ccr2*

**Figure 1:**
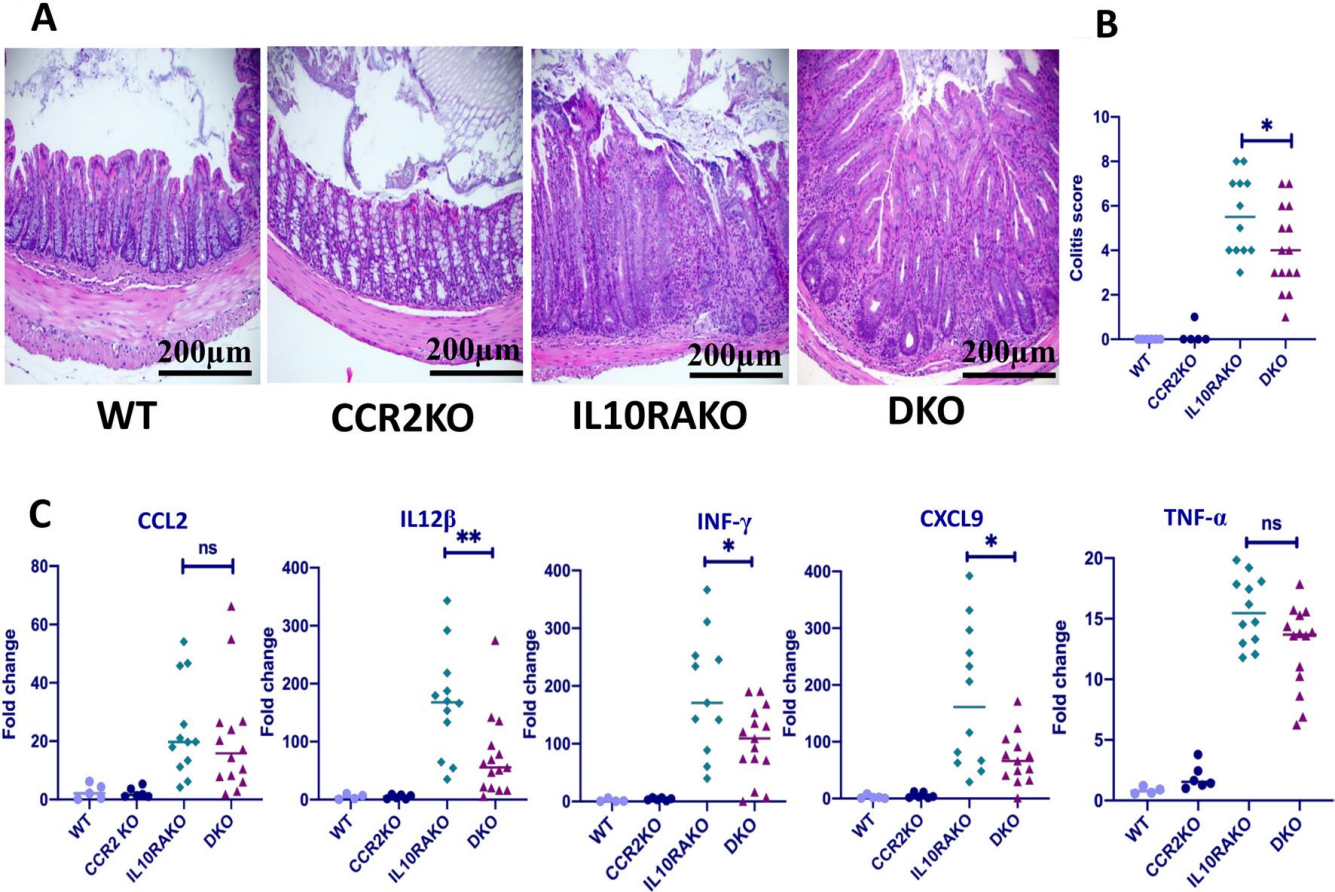
Loss of CCR2 decreases the severity of colitis in IL10RA-deficient mice. (**A**) Representative histological images (H&E) from the colons of 4- to 5-week-old mice of the indicated genotypes (WT = *Cdcs1*^+/+^*Il10ra*^+*/-*^*Ccr2*^+*/-*^; CCR2KO = *Cdcs1*^+*/*+^*Ccr2*^−*/*−^; IL10RAKO = *Cdcs1*^+*/*+^*Il10ra*^−*/*−^; and DKO = *Cdcs1*^+*/*+^*Il10ra*^−*/*−^*Ccr2*^−*/*−^). 20 × magnification, scale bar 200 μm. (**B**) Histology scores (0–15) in groups of WT (n = 4), CCR2KO (n = 6), IL10RAKO (n = 12) and DKO (n = 15) mice. (**C**) Colonic gene expression in mice of the indicated genotype determined by RT-PCR. Fold-change indicates ΔΔCT compared to a selected WT mouse. The results are pooled from 5 separate experiments.

We have shown that the absence of IL10RA leads to the rapid development of infant-onset colitis in C57BL/6 mice that also harbor *Cdcs1*^17^. Colitis in *Cdcs1*^+*/*+^*Il10ra*^−*/*−^ mice is characterized by marked elevation of inflammatory cytokines, including *Il12b, Infg, Il1b and Tnf*^17^*.* While the absence of CCR2 reduces the severity of colitis in DSS-treated mice^25^, it did not appear to reduce the severity of colitis in mice on a mixed 129 and C57BL/6 background that lacked *Il10*^26^. Thus, it remains unclear whether CCR2 function and CCR2-dependent monocyte recruitment are necessary for the development of colitis in mice lacking IL-10R signaling. To address this issue, we compared the severity of colitis in *Cdcs1*^+*/*+^ (C57BL/6) mice lacking either *Il10ra* alone or both *Il10ra* and *Ccr2*. Consistent with our previously reported results, we observed that *Cdcs1*^+*/*+^*Il10ra*^−*/*−^ mice exhibit robust spontaneous colitis by 4–5 weeks of age. Histological assessment of the colon revealed cryptitis with crypt regeneration, distortion, and crypt hyperplasia (Fig. 1A). However, we found that the histological signs of colitis were less severe in *Cdcs1*^+*/*+^ mice that lacked both *Il10ra* and *Ccr2* than in littermates that lacked *Il10ra* alone (Fig. 1A, B). Consistent with reduced histologic signs of colitis, we observed decreased expression of genes associated with pathogenesis of colitis, including *Il12b, Infg*, and the IFNg regulated gene *Cxcl9,* within total RNA isolated from the colon of *Cdcs1*^+*/*+^*Il10ra*^−*/*−^*Ccr2*^−*/*−^ mice compared to expression observed within the colon of *Cdcs1*^+*/*+^*Il10ra*^−*/*−^ mice (Fig. 1C). Furthermore, the median value of *Tnf* expression within the colon of *Cdcs1*^+*/*+^*Il10ra*^−*/*−^*Ccr2*^−*/*−^ mice was lower than that observed in *Cdcs1*^+*/*+^*Il10ra*^−*/*−^ mice, although this difference was not statistically significant. Interestingly,we did not detect difference in the expression of the the CCR2 ligand CCL2 within the colons of *Cdcs1*^+*/*+^*Il10ra*^−*/*−^ and *Cdcs1*^+*/*+^*Il10ra*^−*/*−^*Ccr2*^−*/*−^ mice. Therefore, we suggest that the absence of CCR2 interferes with the development of colitis in mice lacking the receptor for IL10 without altering the expression of its ligand CCL2.

### Reduced accumulation of immature macrophages in *Cdcs1*^+*/*+^*Il10ra*^−*/*−^ mice lacking *Ccr2*

**Figure 2:**
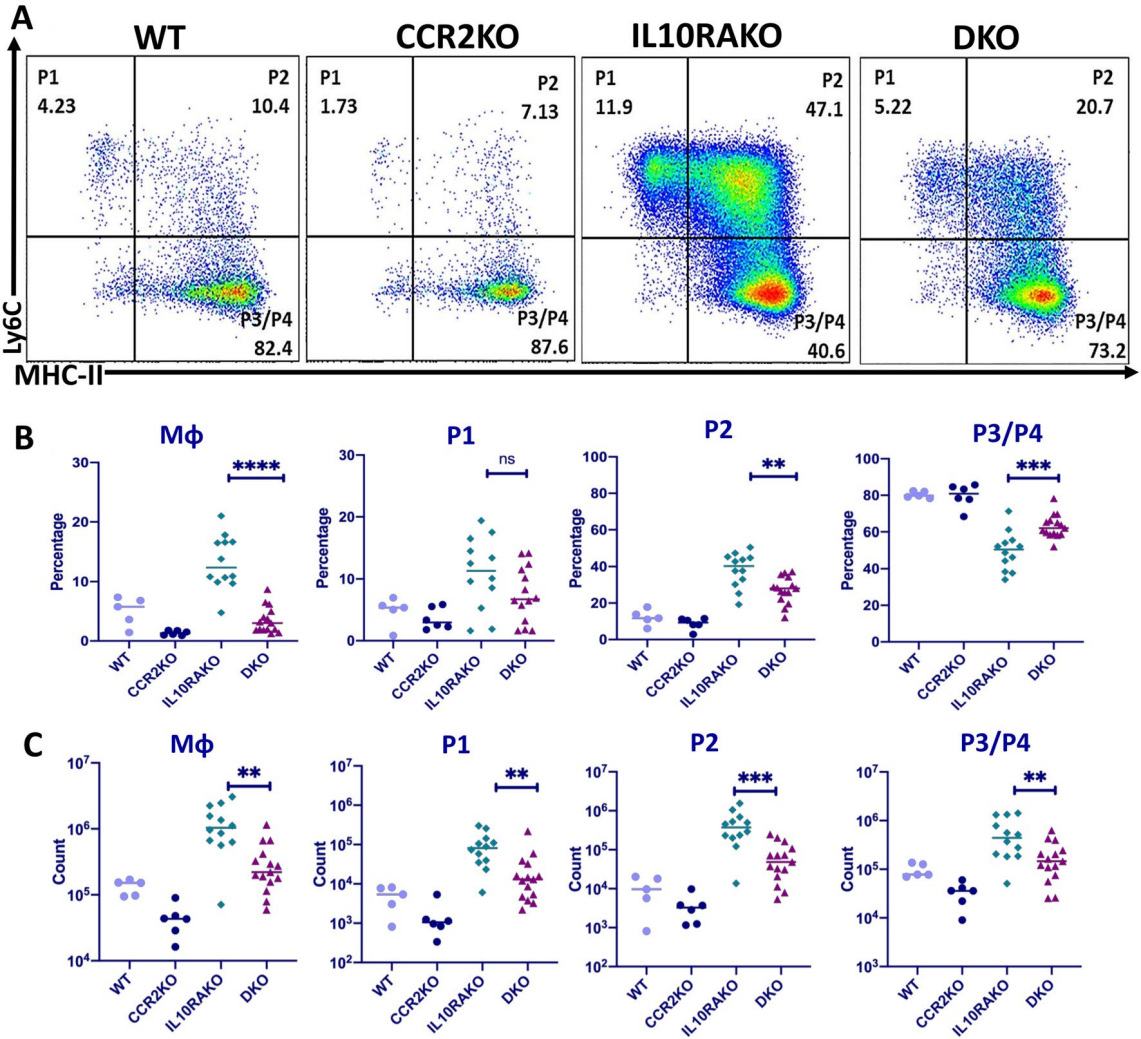
The absence of CCR2 inhibits the accumulation of immature macrophages in the colon of IL10RA-deficient mice. (**A**) Representative plots showing the distribution of P1 (Ly6C^+^MHCII^-^), P2 (Ly6C^+^MHCII^+^) and P3/P4 (Ly6C^-^MHCII^+^) CD11b^+^CD64^+^ lamina propria macrophages as determined by flow cytometry in animals of the indicated genotype. (**B**) Percent and (**C**) Absolute number of LP macrophages in groups of WT (n = 5), CCR2KO (n = 6), IL10RAKO (n = 12) and DKO (n = 15) mice determined by flow cytometry as in (**A**). Please note that the y-axis on the graphs in (**C**) are log scale.

Our data demonstrate that the absence of CCR2 reduces the severity of colitis in mice lacking IL-10R signaling. As prior data have demonstrated that CCR2 is necessary for monocyte recruitment into the healthy colon^19^, we wondered whether the reduced severity of colitis observed in mice lacking both CCR2 and IL10R was associated with reduced accumulation of macrophages. To assess this possibility, we isolated colonic LP macrophages from 4- to 5-week-old *Cdcs1*^+*/*+^*Il10ra*^−*/*−^ and *Cdcs1*^+*/*+^*Il10ra*^−*/*−^*Ccr2*^−*/*−^ mice and analyzed their phenotype by flow cytometry. The gating strategy for these experiments is shown in supplemental (Fig. S1). We found a significant decrease in the frequency of colonic CD11b^+^CD64^+^ macrophages within the total CD45^+^ cell population of *Cdcs1*^+*/*+^*Il10ra*^−*/*−^*Ccr2*^−*/*−^ mice compared to *Cdcs1*^+*/*+^*Il10ra*^−*/*−^ mice (Fig. 2A,B), and this translated into a significant decrease in the total number of CD11b^+^CD64^+^ macrophages in the colon of *Cdcs1*^+*/*+^*Il10ra*^−*/*−^*Ccr2*^−*/*−^ mice compared to the numbers found in the colons of *Cdcs1*^+*/*+^*Il10ra*^−*/*−^ mice (Fig. 2C). Furthermore, we observed that the absolute number of immature Ly6C^+^ macrophages (both MHCII^−^ (P1) and MHCII^+^ (P2)) within the total CD11b^+^CD64^+^ macrophage population was significantly decreased in *Cdcs1*^+*/*+^*Il10ra*^−*/*−^*Ccr2*^−*/*−^ mice compared to *Cdcs1*^+*/*+^*Il10ra*^−*/*−^ mice (Fig. 2C), and this was accompanied by a decrease in the proportion of both the P1 and P2 populations in *Cdcs1*^+*/*+^*Il10ra*^−*/*−^*Ccr2*^−*/*−^ mice, although this only reached statistical significance for the P2 population (Fig. 2B). Interestingly, we noted a significant increase in the frequency of mature Ly6C^−^MHCII^+^ (P3/P4) macrophages within the total CD11b^+^CD64^+^ macrophage population of *Cdcs1*^+*/*+^*Il10ra*^−*/*−^*Ccr2*^−*/*−^ mice compared to the proportion observed in *Cdcs1*^+*/*+^*Il10ra*^−*/*−^ mice, although this was not reflected by an increase in the absolute number of P3/P4 populations in these mice, likely because the total number of macrophages in the colon of these mice was reduced compared to *Cdcs1*^+*/*+^*Il10ra*^−*/*−^ mice. Importantly, we did not detect significant decrease in frequency and numbers of colonic Ly6G^+^ neutrophils between *Cdcs1*^+*/*+^*Il10ra*^−*/*−^*Ccr2*^−*/*−^ and *Cdcs1*^+*/*+^*Il10ra*^−*/*−^ mice (Fig. S2). Thus, our data demonstrate that the reduced severity of colitis observed in mice lacking both IL-10RA and CCR2 is associated with a decrease in the accumulation of colonic macrophages and a decrease in the proportion of immature macrophages observed within this population.

### Reduced frequency of classical blood monocytes in *Cdcs1*^+*/*+^*Il10ra*^−*/*−^ deficient mice lacking *Ccr2*

**Figure 3:**
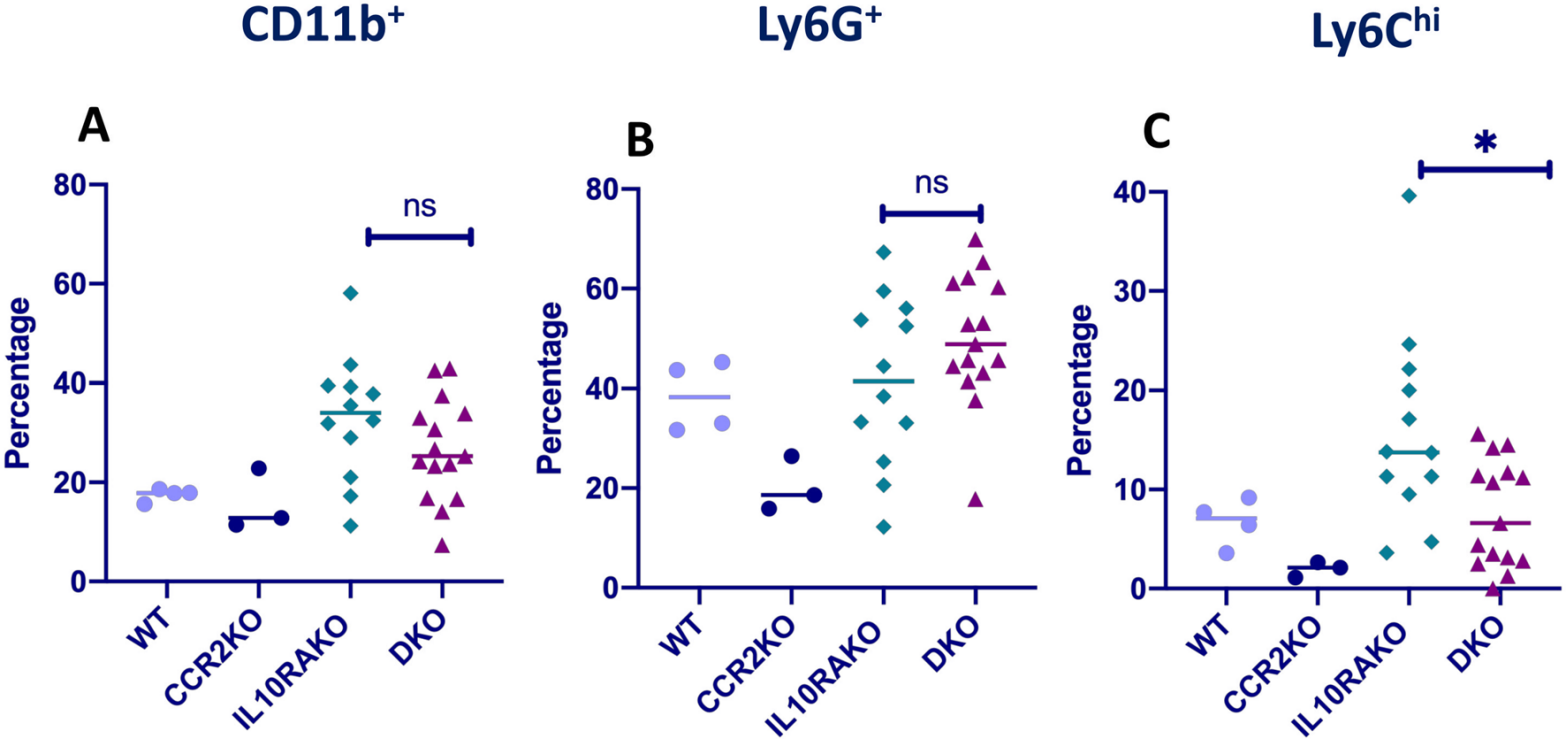
Decreased circulating Ly6C^hi^ monocytes in IL10RA-deficient mice lacking CCR2. The percentages of (**A**) CD11b^+^ cells within the CD45^+^gate, (**B**) Ly6G^+^, and (**C**) Ly6C^hi^ cells within the CD11b^+^gate of circulating blood cells isolated from WT (n = 4), CCR2KO (n = 3), IL10RAKO (n = 12) and DKO (n = 15) mice, as determined by flow cytometry.

It has been previously demonstrated that *Ccr2*-deficient mice exhibit a decreased frequency of Ly6C^hi^ monocytes in the circulation and reciprocal increases within the bone marrow, suggesting that in addition to a potential role in directing monocyte recruitment from the blood to the colon, CCR2 is necessary for efficient emigration of Ly6C^hi^ monocytes from the bone marrow to the blood^19,23,27^. We wondered whether a decreased frequency of circulating monocytes in *Cdcs1*^+*/*+^*Il10ra*^−*/*−^*Ccr2*^−*/*−^ mice compared to *Cdcs1*^+*/*+^*Il10ra*^−*/*−^ mice could be a factor contributing to the decreased number and frequency of immature Ly6C^+^ macrophages observed in the colon of *Cdcs1*^+*/*+^*Il10ra*^−*/*−^*Ccr2*^−*/*−^ mice. Therefore, we compared the percentages of CD11b^+^Ly6C^+^ monocytes in the blood of *Cdcs1*^+*/*+^*Il10ra*^−*/*−^ and *Cdcs1*^+*/*+^*Il10ra*^−*/*−^*Ccr2*^−*/*−^ mice using flow cytometry (gating stategy shown in Fig. S3). Although we did not detect a significant difference in the frequency of total blood CD11b^+^ cells or CD11b^+^Ly6G^+^ neutrophils, we observed a significant decrease in the frequency of circulating Ly6C^hi^ monocytes in *Cdcs1*^+*/*+^*Il10ra*^−*/*−^*Ccr2*^−*/*−^ mice compared to *Cdcs1*^+*/*+^*Il10ra*^−*/*−^ mice (Fig. 3A–C). This raises the possibility that the reduced accumulation of immature macrophages within the colon of *Cdcs1*^+*/*+^*Il10ra*^−*/*−^*Ccr2*^−*/*−^ mice may in part be explained by the reduced frequency of circulating monocytes.

### Reduction in Iba1-positive cells within the colon of *Cdcs1*^+*/*+^*Il10ra*^−*/*−^*Ccr2*^−*/*−^ mice

**Figure 4:**
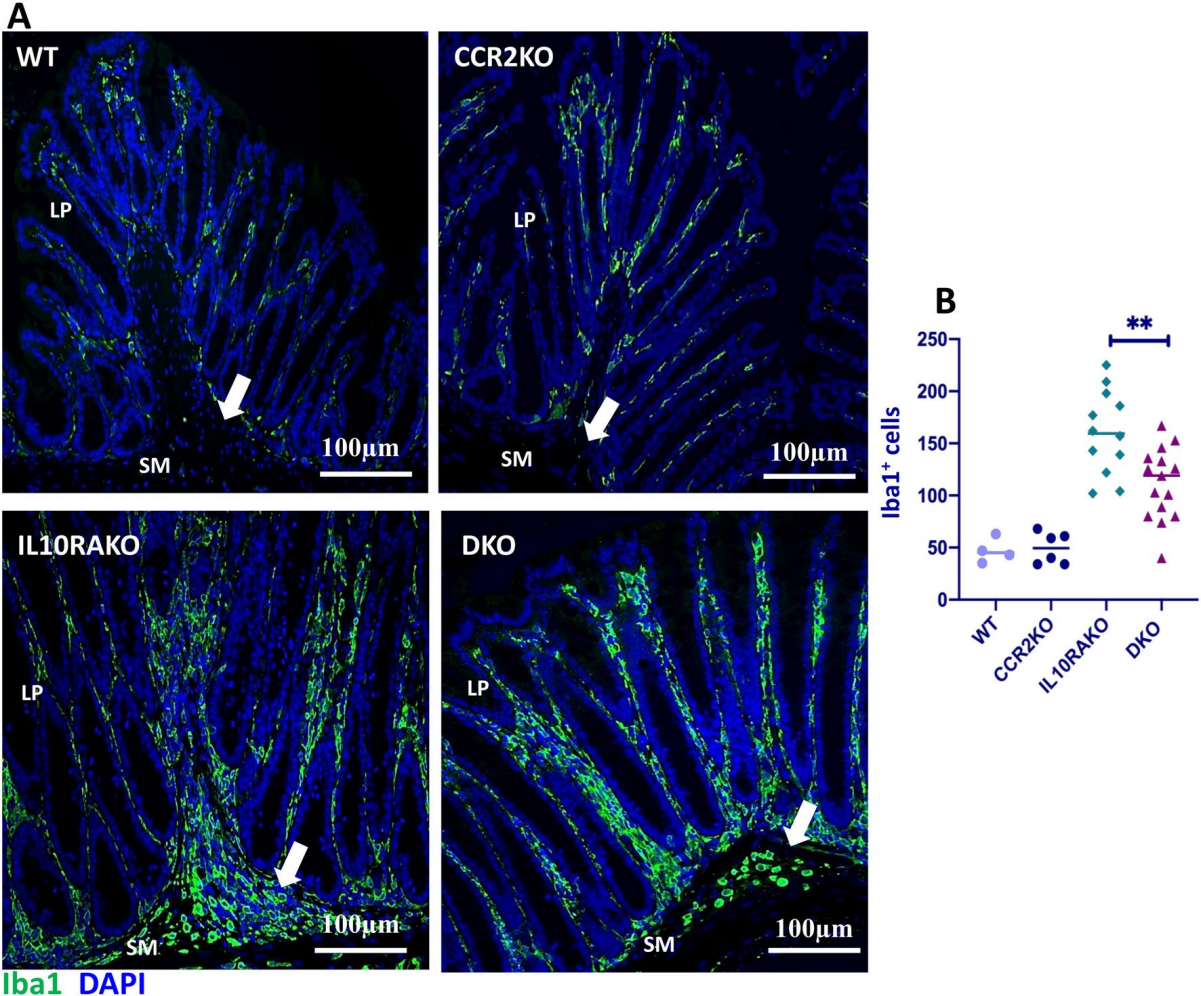
CCR2 facilitates the accumulation of round macrophages within the colon submucosa of IL10RA-deficient mice. (**A**) Immunofluorescent images from the proximal colon of representative mice of the indicated genotypes showing the localization and morphology of cells staining with Iba1. 20 × magnification, Scale bar indicates 100 μm. The images from IL10RAKO and DKO mice demonstrated a similar density of ramified Iba1-positive cells in the lamina propria (LP) but a reduction in round Iba1-positive cells within the submucosa (SM, filled arrows) of DKO mice compared to IL10RAKO mice. (**B**) Numbers of Iba1-positive cells in 5 fields from the colon of WT (n = 4), CCR2KO (n = 6), IL10RAKO (n = 12) and DKO (n = 15) mice, as indicated.

Mature Ly6C^−^ macrophages have previously been described within the lamina propria (LP), submucosa, and muscularis layers of the colon^28^. Immature Ly6C^+^ macrophages have been described within the LP of IL10R-deficient mice with colitis^17,22^, but whether immature Ly6C^+^ macrophages are found in other layers within the colon of IL10R-deficient mice with colitis has not been thoroughly described. Therefore, we used immunofluorescent staining with an Iba1 antibody, a commonly used marker to identify resident macrophage populations in the intestine^29^, to compare the localization and shape of macrophages within the colons of *Cdcs1*^+*/*+^*Il10ra*^+*/−*^, *Cdcs1*^+*/*+^*Ccr2*^−*/*−^*, Cdcs1*^+*/*+^*Il10ra*^−*/*−^, and *Cdcs1*^+*/*+^*Il10ra*^−*/*−^*Ccr2*^−*/*−^ mice. In addition to a population of large, ramified cells present within the LP of both *Cdcs1*^+*/*+^*Il10ra*^+*/-*^ and *Cdcs1*^+*/*+^*Ccr2*^−*/*−^ mice, we observed round Iba1-positive cells predominantly localized within the submucosa of *Cdcs1*^+*/*+^*Il10ra*^−*/*−^ mice that were largely absent from wild-type mice or those lacking CCR2 alone (Fig. 4A). As it has been previously demonstrated that monocyte-derived macrophages transition from a round to ramified shape upon recruitment to the colon^30^, these results raised the possibility that immature macrophages selectively accumulated in the submucosa of *Cdcs1*^+*/*+^*Il10ra*^−*/*−^ mice. While it appeared that the density of round Iba1-positive cells in *Cdcs1*^+*/*+^*Il10ra*^−*/*−^*Ccr*2^−*/*−^ mice was reduced compared to the density observed in *Cdcs1*^+*/*+^*Il10ra*^−*/*−^ littermate controls (arrows in Fig. 4A), the density of the Iba1^+^ ramified cells within the LP of both genotypes seemed largely preserved. Manual quantification of the total number of Iba1^+^ cells in the lamina propria and submucosa demonstrated a significant decrease in total Iba1-positive cells in *Cdcs1*^+*/*+^*Il10ra*^−*/*−^*Ccr2*^−*/*−^ mice compared to *Cdcs1*^+*/*+^*Il10ra*^−*/*−^ mice (Fig. 4B). Taken together with our flow cytometric results, these observations suggest that the round Iba1^+^ cells observed within the submucosa of *Cdcs1*^+*/*+^*Il10ra*^−*/*−^ mice correspond to recently recruited immature macrophages and that this recruitment depends at least in part upon the presence of CCR2.

### The accumulation of rounded Iba1^+^ macrophages is the result of a cell-intrinsic loss of IL10RA

**Figure 5:**
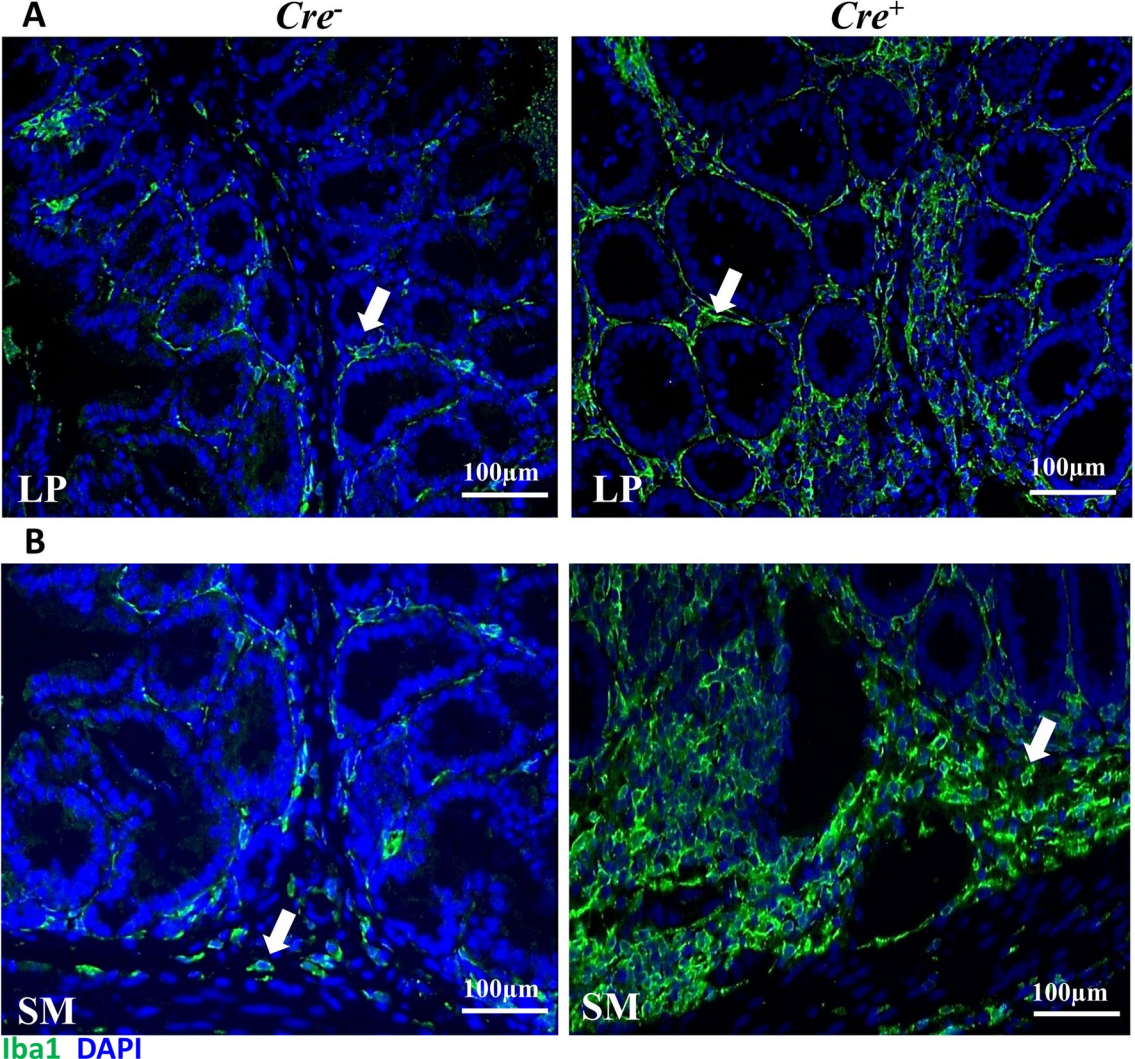
Intrinsic IL10R signaling prevents the accumulation of round macrophages within the colonic submucosa. (**A**) Immunofluorescent images from the colon of representative *Cdcs1*^+*/*+^*Il10ra*^*fl/fl*^*LysMCre*^*−*^ (Cre^−^) and *Cdcs1*^+*/*+^*Il10ra*^*fl/fl*^*LysMCre*^+^ (Cre^+^) mice showing the ramified shaped Iba1^+^ cells within the colonic lamina propria (LP) indicated by the arrows. (**B**) Increased density of Iba1^+^ round shaped cells (arrows) within the submucosa (SM) of Cre^+^ mice compared to Cre^-^ mice. 20 × magnification, Scale bar indicates 100 μm.

We have previously demonstrated that mice specifically lacking IL10RA in myeloid cells (*Cdcs1*^+*/*+^*Il10ra*^*fl/fl*^*LysM*^*Cre*+^) demonstrate accumulation of immature Ly6C^+^ macrophages within the lamina propria^17^, but whether this is associated with the accumulation of rounded Iba1^+^ macrophages within the submucosa was not previously evaluated. To address this, we compared Iba1 staining within colonic sections obtained from control (*Cdcs1*^+*/*+^*Il10ra*^*fl/fl*^*LysM*^*Cre−*^) and *Cdcs1*^+*/*+^*Il10ra*^*fl/fl*^*LysM*^*Cre*+^ mice. Interestingly, we observed ramified Iba1^+^ cells within the LP, as well as a high density of Iba1^+^ round shaped cells within the submucosa of *Cdcs1*^+*/*+^*Il10ra*^*fl/fl*^*LysM*^*Cre*+^ mice, which were uncommon in the control mice (Fig. 5A,B). These data are consistent with the concept that intrinsic IL10R signaling on immature macrophages interferes with the CCR2-dependent accumulation of these cells in the intestinal submucosa.

### The absence of CCR2 does not alter proinflammatory gene expression in mature IL10RA-deficient colonic macrophages

**Figure 6:**
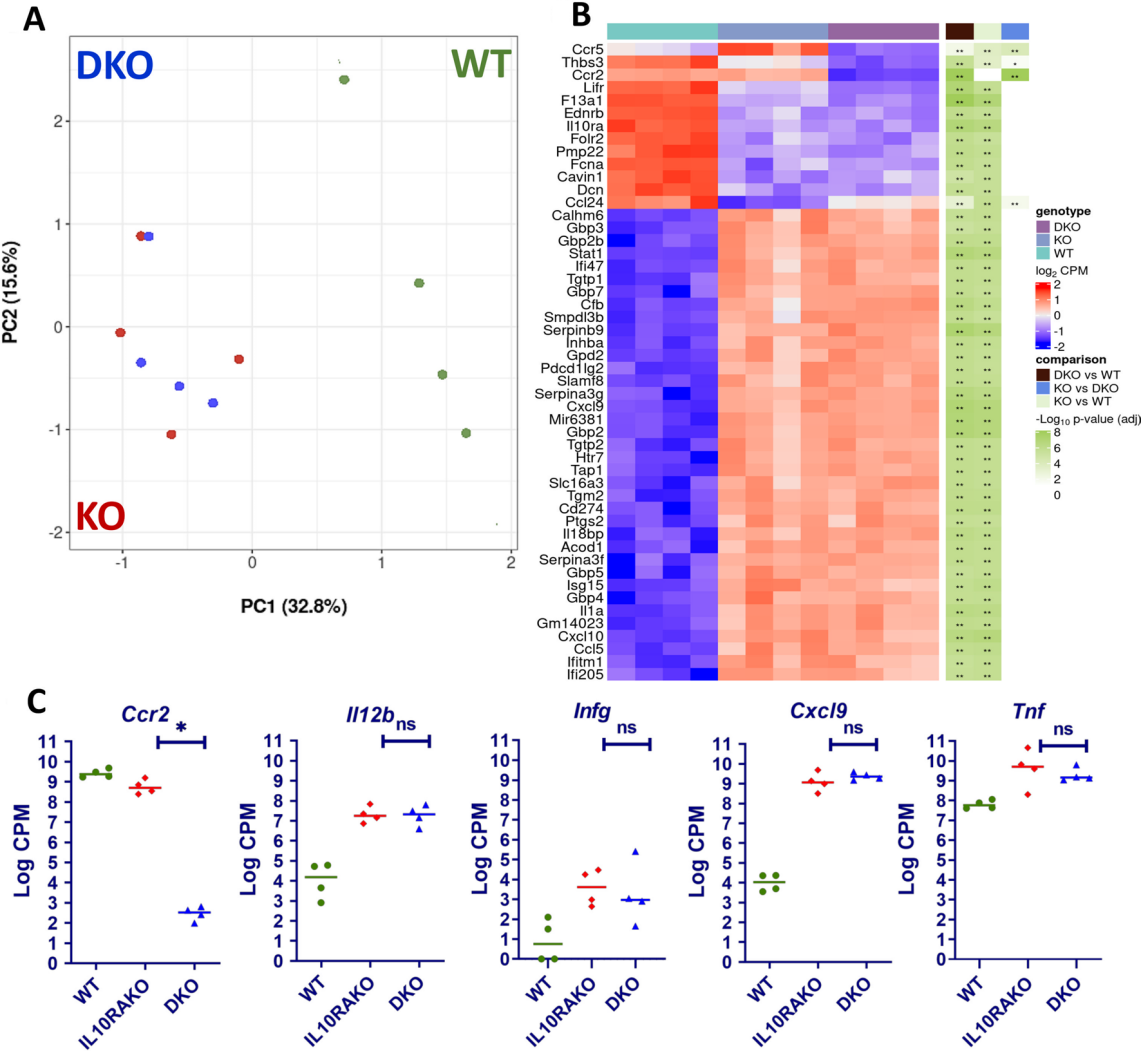
The absence of CCR2 does not change the gene expression signature of IL10RA-deficient intestinal macrophages. (**A**) Principal component analysis comparing the gene expression profiles of MHCII^+^Ly6C^−^ colonic macrophages isolated from WT (*Cdcs1*^+*/*+^), IL10RAKO (*Cdcs1*^+*/*+^*Il10ra*^−*/*−^), and DKO (*Cdcs1*^+*/*+^*Il10ra*^−*/*−^*Ccr2*^−*/*−^) mice determined by RNAseq. (**B**) Heat map demonstrating differences in gene expression between groups, including the 4 genes that were expressed at significantly different levels between macrophages isolated from IL10RAKO (KO) and DKO mice, as well as an additional 46 genes that with the lowest adjusted *P* values between WT and IL10RAKO macrophages. (**C**) Expression of selected genes as indicated. WT (n = 4), IL10RAKO (n = 4) and DKO (n = 4) mice. Log CPM = Log2 counts per million reads.

The experiments described above suggested that the absence of CCR2 interferes with the development of inflammation in IL10RA-deficient mice by limiting the accumulation of immature macrophages within the submucosal layer of the intestine. However, whether the absence of CCR2 in colonic macrophages also has independent effects on proinflammatory gene expression in these cells remains to be determined. To address this issue, we isolated mature MHCII^+^Ly6C^−^ macrophages from 4- to 5-week-old *Cdcs1*^+*/*+^*, Cdcs1*^+*/*+^*Il10ra*^−*/*−^*,* and *Cdcs1*^+*/*+^*Il10ra*^−*/*−^*Ccr2*^−*/*−^ mice using fluorescence-activated cell sorting and compared gene expression in these populations by RNA sequencing. Interestingly, principal component analysis (PCA) demonstrated that mature Ly6C^−^ macrophages in both *Cdcs1*^+*/*+^*Il10ra*^−*/*−^*Ccr2*^−*/*−^ and *Cdcs1*^+*/*+^*Il10ra*^−*/*−^ mice clustered closely together in a position that was distinct from *Cdcs1*^+*/*+^ macrophages (Fig. 6A). Although as expected CCR2 expression was absent in *Cdcs1*^+*/*+^*Il10ra*^−*/*−^*Ccr2*^−*/*−^ macrophages, we were only able to identify 3 other genes that were expressed at significantly different levels between macrophages from *Cdcs1*^+*/*+^*Il10ra*^−*/*−^*Ccr2*^−*/*−^ and *Cdcs1*^+*/*+^*Il10ra*^−*/*−^, despite significant differences in expression between many genes in *Cdcs1*^+*/*+^ and *Cdcs1*^+*/*+^*Il10ra*^−*/*−^ colonic macrophages (Fig. 6B). Further, in directed comparisions we were unable to identify significant differences in the expression of several key inflammatory regulators, including *Il12b*, *Infg, Tnf,* and *Cxcl9*, between *Cdcs1*^+*/*+^*Il10ra*^−*/*−^ and *Cdcs1*^+*/*+^*Il10ra*^−*/*−^*Ccr2*^−*/*−^ macrophages (Fig. 6C). These results indicate that the absence of CCR2 has little effect on the induction of key inflammatory genes observed in mature intestinal macrophages lacking IL10RA. These data are consistent with the hypothesis that reduced inflammation observed in IL10R-deficient mice that also lack CCR2 is primarily the result of decreased macrophage accumulation in the colon following an inflammatory stimulus rather than a primary defect in the ability of CCR2-deficient intestinal macrophages to produce inflammatory cytokines.

## Discussion

IBD is a multifactorial disease involving microbial, environmental, and genetic components^31^. Rare loss-of-function mutations in IL10R are associated with the development of IBD during infancy^9^. Similarly, we have shown that mice lacking IL10R develop early-onset spontaneous colitis at 3–4 weeks of age on a genetically susceptible *Cdcs1* background^17^. We and others have shown that mice with colitis caused by the absence of IL10R or that induced by treatment with DSS demonstrate marked increases in the accumulation of immature Ly6C^+^ macrophages within the colon^10,15,17,25^. While these macrophages are thought to play a central role in disease pathogenesis observed in mice lacking IL10R, the factors involved in the accumulation of these immature macrophages within the colon remain to be completely defined. Here, we have shown that the absence of the chemokine receptor CCR2 interferes with colitis development in IL10R-deficient mice, as demonstrated by reduced colitis scores and decreased expression of genes associated with disease severity, including *Il12b*, *Infg*, and the INFg-induced gene *Cxcl9,* in mice lacking both IL10RA and CCR2 compared to mice lacking IL10RA alone. These data are consistent with a prior report of reduced pathology in DSS-treated CCR2-deficient mice^25^. In contrast, it has previously been reported that the absence of CCR2 in IL10-deficient mice did not influence colitis severity^26^. Although our model incorporated the absence of IL10R rather than the absence of IL10, it is not initially clear why our results differ from those of this previous study. These differences may reflect differences in the genetic background used in these two studies. Whereas the prior study employed mice on a mixed 129 and C57BL/6 background, which was apparently susceptible to colitis caused by the absence of IL10, our study used C57BL/6 mice, which were congenic for the *Cdcs1* IBD susceptibility loci. We have previously demonstrated that a variant of *Alpk1* in the *Hiccs* loci is associated with disease susceptibility on the *Cdcs1* background, but whether genetic background is the primary factor influencing the difference between these two animal models remains to be determined^32^.

The reduced severity of colitis observed in IL10R-deficient mice lacking CCR2 was accompanied by a significant reduction in the number and frequency of immature Ly6C^+^ colonic macrophages compared to mice lacking IL10R alone. A reduction in the frequency of TLR2^+^F4/80^+^BrdU^+^ macrophages (markers associated with immature macrophages) was observed in DSS-treated CCR2-deficient mice, while a reduction in the accumulation of colonic macrophages was not observed in *Il10*^−*/*−^*Ccr2*^−*/*−^ mice^25,26^, although immature and mature macrophages were not distinguished in the latter study. In our study, the most profound reductions were found within the immature Ly6C^+^ colonic macrophage populations, and thus, it is possible that these differences may not have been detected in the study using *Il10*^−*/*−^*Ccr2*^−*/*−^ mice. Interestingly, using immunofluorescence, we demonstrated a reduction in Iba1^+^ round shaped cells within the colonic submucosa, while Iba1^+^ ramified cells were preserved within the lamina propria. Iba1^+^ identifies monocytes and microglia in the brain and resident macrophages in the muscularis mucosa^29,33^. We suggest that Iba1 staining also identifies newly recruited immature macrophages in the submucosa as well as mature macrophages in the lamina propria, and that the reduction in Iba1^+^ round shaped cells in the submucosa of *Il10*^−*/*−^*Ccr2*^−*/*−^ mice is consistent with a defect in recruitment of immature macrophages into colonic submucosa in the absence of CCR2. Our results are consistent with a recent study demonstrating a reduction in the accumulation of round-shaped immature intestinal macrophages following reconstitution of sublethally irradiated mice with CCR2-deficient bone marrow cells^30^.

Although we demonstrated a reduction in the accumulation of immature macrophages in *Cdcs1*^+*/*+^*Il10ra*^−*/*−^*Ccr2*^−*/*−^ mice compared to *Cdcs1*^+*/*+^*Il10ra*^−*/*−^ mice, the basis for this reduction remains unclear. It has previously been suggested that CCR2 is necessary for the migration of monocytes from the bone marrow to the circulation based on decreased numbers of circulating monocytes and increased numbers of monocytes within the bone marrow of CCR2-deficient mice^23^. In our study, we also demonstrated a reduced frequency of circulating monocytes in *Cdcs1*^+*/*+^*Il10ra*^−*/*−^*Ccr2*^−*/*−^ mice compared to *Cdcs1*^+*/*+^*Il10ra*^−*/*−^ mice, suggesting that even under inflammatory conditions, the absence of CCR2 reduced the frequency of circulating monocytes. It thus seems possible that a reduction in circulating monocytes in *Cdcs1*^+*/*+^*Il10ra*^−*/*−^*Ccr2*^−*/*−^ mice could be a factor that limits the accumulation of immature monocytes in the colon under inflammatory conditions.

An alternative possibility is that CCR2 has a direct role in orchestrating the recruitment of monocytes from the circulation into the colon. Prior studies have demonstrated an impairment in the accumulation of *Ccr2*^−*/*−^ monocytes into the colon and small intestinal lamina propria compared to wild-type monocytes when an equal mixture of both were transferred into CCR2-deficient mice^34^, although whether this was independent of observed increases in sequestration within the bone marrow was not completely defined. Another study demonstrated that following sublethal irradiation, round CCR2^hi^CX3CR1^+^ monocytes derived from donor bone marrow cells accumulate in the colon lamina propria and undergo a phenotypic shift toward more elongated CX3CR1 + macrophages over the ensuing 2 weeks^30^. Despite this evidence that CCR2 may play an important role in recruitment into the lamina propria, our observations that increased numbers of immature monocytes accumulate within the colon of inflamed *Cdcs1*^+*/*+^*Il10ra*^−*/*−^*Ccr2*^−*/*−^ mice compared to *Cdcs1*^+*/*+^*Ccr2*^−*/*−^ mice suggest that CCR2 is not absolutely required for the recruitment of blood monocytes into the inflamed colon. Indeed, it has previously been suggested that other chemokine receptors, including CCR1, may also have important roles in monocyte recruitment into the colon^35^. Thus, the relative contributions of defective recruitment and decreased numbers of circulating monocytes to the reduced accumulation of immature monocytes observed within the colon of *Cdcs1*^+*/*+^*Il10ra*^−*/*−^*Ccr2*^−*/*−^ mice remain to be completely defined.

In addition to influencing the accumulation of immature monocytes in the inflamed colon, it is also possible that CCR2 signaling could have a direct role in regulating the magnitude of their responses to inflammatory stimuli. We found minimal differences in inflammatory gene expression of sorted mature Ly6C^−^MHCII^+^ macrophages isolated from the colon of mice lacking IL10R alone and those lacking both IL10R and CCR2. Thus, we suggest that the primary mechanism that leads to decreased inflammation in *Il10ra*^−*/*−^*Ccr2*^−*/*−^ mice is reduced accumulation of immature macrophages.

Although we demonstrated reduced accumulation of immature macrophages and reduced severity of colitis in *Cdcs1*^+*/*+^*Il10ra*^−*/*−^*Ccr2*^−*/*−^ mice compared to *Cdcs1*^+*/*+^*Il10ra*^−*/*−^ mice, *Cdcs1*^+*/*+^*Il10ra*^−*/*−^*Ccr2*^−*/*−^ mice continued to develop colitis of moderate severity. Colitis scores in *Cdcs1*^+*/*+^*Il10ra*^−*/*−^*Ccr2*^−*/*−^ mice are significantly higher than either wild-type mice or those that lack CCR2 alone (*Cdcs1*^+*/*+^), which do not develop histologic signs of colitis, and there are likewise significant increases in the number of colonic macrophages isolated from *Cdcs1*^+*/*+^*Il10ra*^−*/*−^*Ccr2*^−*/*−^ mice compared to these control strains. Thus, CCR2 is not absolutely required either for the development of inflammation or the accumulation of immature colonic macrophages in mice lacking IL10R. Prior studies, including those from our laboratory, have emphasized the importance of colonic macrophages in driving inflammation in mice lacking IL10R signaling^16–18^, suggesting potentially important roles of CCR2-independent colonic macrophage populations. CCR2-independent populations could arise from blood monocytes that are recruited in a CCR2-independent fashion or, alternatively, could be derived from long-lived resident colonic macrophage populations rather than blood monocytes. Interestingly, it has been suggested that mature colonic macrophages in adult mice originate both from embryonic precursors and blood monocytes^19,28,36^. Prior studies have identified three ontogenetically distinct macrophage populations defined by the expression of CD4 and Tim4 that exist within the Ly6C^−^ mature macrophage populations of both the large and small bowel. Tim4^+^CD4^+^ macrophages exhibit gene signatures associated with tissue residency, and they were the predominant species found within the Ly6C^−^MHCII^+^ fraction of intestinal macrophages in both wild-type and *Ccr2*^−*/*−^ mice in infancy and adulthood. In contrast, Tim4^-^ (CD4^+^ and CD4^−^) only accumulated within intestinal tissues later during adulthood^35^, and the accumulation of this subset was impaired in *Ccr2*^−*/*−^ mice^36^. Thus, our observation that mature macrophage populations persist in *Cdcs1*^+*/*+^*Il10ra*^−*/*−^*Ccr2*^−*/*−^ mice despite reduced accumulation of immature macrophages suggests that either these macrophages are primarily of embryonic origin, or alternatively, despite the reduction in accumulation of immature macrophages, the mature macrophage niche can be filled by monocyte-derived macrophages in a CCR2-independent fashion. Such a scenario would be consistent with studies suggesting that mature colonic macrophages have a considerably longer life span than immature macrophages. Future studies will be necessary to untangle the origin of these mature colonic macrophage populations in *Cdcs1*^+*/*+^*Il10ra*^−*/*−^*Ccr2*^−*/*−^ mice and to understand their role in driving inflammation in the absence of IL10R signaling.

## Materials and methods

### Mice

*Cdcs1*^+*/*+^*II10ra*^−*/*−^*Ccr2*^−*/*−^ mice were generated by crossing *Ccr2*^−*/*−^ mice (B6.129S4-*Ccr2*^*tm1Ifc*^/J_004999) (The Jackson Laboratory, Bar Harbor, ME)^37^ with *Cdcs1*^+*/*+^*Il10ra*^−*/*−^ mice^17^. *Cdcs1*^+*/*+^*Il10ra*^*fl/fl*^*LysMCre*^*−*^ and *Cdcs1*^+*/*+^*Il10ra*^*fl/fl*^*LysMCre*^+^ mice were generated as we have previously described^17^. All mice were housed in a specific pathogen-free animal facility at Boston Children’s Hospital. Controls consisted of cohoused littermates of the appropriate genotype. Both male and female mice were used throughout this study. All mice were between 28–35 days old when analyzed. All experiments were conducted following approval from the Animal Resources at Children’s Hospital, per regulations of the Institutional Animal Care and Use Committees (IACUC, Assurance number: A3303-01).

### Reagents and antibodies

Reagents used for flow cytometry included Zombie VioletTM fixable viability kit (BioLegend, San Diego, CA) and the following anti-mouse antibodies: CD16/32 (clone 93, Fc blocking, BioLegend), Bv605-CD45 (clone 30-F11, BioLegend), PE. Cy7-CD11b (clone M1/70, BioLegend), APC-CD64 (clone X54-5/7.1, BioLegend), FITC-I-A/I-E (MHCII, clone M5/114.15.2, BioLegend), APC. Cy7-Ly6C (clone HK1.4, BioLegend), Bv711-CD3e (clone 145–2 C11, BioLegend), and Percp/Cy5.5-Ly6G (clone IA8, Biolegend). Immune fluorescence staining was performed using the primary antibody Iba1 (rabbit; Wako Cat# 019-1941, RRID: AB_2313566) and secondary goat anti-rabbit antibody; Alexa Fluor-488, Invitrogen).

### Disease severity scoring

Colons from mice were collected and divided into proximal, middle and distal colonic segments. The tissues were fixed in 10% neutral buffered formalin for 24–48 h, stained with H&E, and scored in a blinded fashion by a board-certified pathologist (J.N.G) using the scoring criteria previously described^38,39^. Briefly, scores represent the sum of subscores (0–3) for the following criteria: mononuclear inflammation (exclusive of normal GALT and lymphoid aggregates), crypt hyperplasia, epithelial injury, and neutrophilic inflammation/crypt abscesses. Images were acquired using a brightfield light ordinary microscope under 20 × magnification.

### Isolation of LP cells and flow cytometry

Colonic lamina propria cells were isolated as we have previously described^17^. Briefly, colons were dissected, and epithelial cells were stripped by agitation in 10 mM EDTA for 30 min at 37 °C. The remaining tissue was transferred to 50 ml conical tubes and digested using collagenase VIII (Sigma-Aldrich, C2139-5G) in HBSS (with Ca/Mg) for 30–45 min at 37 °C. Cells were then filtered through 70 µm cell strainers and washed twice in 1 × PBS. Cell viability and numbers were determined using an automated cell counter (TECAN), stained with antibody cocktails as indicated and analyzed using an LSRFortessa (BioLegend, San Diego, CA, USA). Data were analyzed by Flow Jo V.10 software.

### Quantitative real-time PCR

Total RNA was extracted from whole colon tissues using TRIzol (Invitrogen, Grand Island, NY) according to the manufacturer’s instructions. cDNA was synthesized using TaqMan Reverse Transcription Kits and random hexamer primers (Applied Biosystem, N808-0234). RT-PCR was performed using a QuantStudio 6 System with TaqMan probes or SsoAdvanced Universal SYBR Green Supermix (Bio-Rad, 1725274). Primer sequences are available on request. Expression was normalized to beta actin, and differences between samples were calculated using the ΔΔ cycle threshold method. The fold change in gene expression compared to controls was graphed.

### RNA sequencing and computational analysis

A total of 7,000–50,000 LP MFs (P3/P4 fraction) from 5-week-old *Cdcs1*^+*/*+^ (WT), *Cdcs1*^+*/*+^*Il10ra*^−*/*−^ (IL10RAKO) and *Cdcs1*^+*/*+^*Il10ra*^−*/*−^*Ccr2*^−*/*−^ (DKO) mice were FACS-sorted (gates: CD45^+^, CD103^-^ Ly6G^-^, CD64^+^CD11b^+^, Ly6C^-^MHCII^+^) directly in 350 µl RLT lysis buffer (Qiagen, Waltham, MA), and mRNA was extracted using the Qiagen RNeasy Micro kit (50) (Qiagen, 74004, Waltham, MA). Oligo dT beads were used to enrich for mature transcripts. cDNA was synthesized using Takara SmartSeq v4 reagents from 1 ng of RNA. Full-length cDNA was fragmented to a mean size of 200 bp with a Covaris M220 ultrasonicator, and Illumina sequencing libraries were prepared from 2 ng of sheared cDNA using Swift 2S Acel reagents on a Beckman Coulter Biomek i7 according to the manufacturer’s protocol. The finished dsDNA libraries were quantified by a Qubit fluorometer, Agilent TapeStation 2200, and RT-qPCR using the Roche Kapa library quantification kit. Uniquely dual indexed libraries were pooled in equimolar ratios and evaluated for cluster efficiency and pool balance with shallow sequencing on an Illumina MiSeq. Final sequencing was performed on an Illumina NovaSeq6000 with paired-end 150 bp reads at the Center for Functional Cancer Epigenetics, Molecular Biology Core Facility (MBCF) of Dana-Farber Cancer Institute, Boston. For RNAseq analysis, Fastq files were generated using Illumina bcl2fastq v2.20 software. Sequenced reads were aligned to the UCSC mm10 reference genome assembly, and raw gene counts were quantified using STAR (v2.5.1b)^40^. Genes names were harmonized using bioMART R API. Gene counts were normalized and tested for differential expression using edgeR (v3.34.0)^41,42^. Visualizations were created by R packages ggplot2 (v3.3.5) and Enhanced Volcano (v1.10.0). All sequencing data have been deposited in NCBI’s Gene Expression Omnibus and are accessible through GEO Series accession number GSE183960.

### Immunofluorescence staining

Immunofluorescence staining was performed as we have previously reported^43^. In brief, colonic sections were baked at 60 °C overnight, followed by deparaffinization using xylene and rehydration steps through decreasing grades of ethanol (100% to 50%). Antigen retrieval was performed by immersing slides in Tris–EDTA (pH 9.0) at 85 °C for 20 min. Tissue permeabilization was performed using acetone for 10 min followed by three washes in 1 × PBS. Sections were then blocked with blocking buffer containing 5% normal goat serum, 5% BSA (IgG free; protease free, Jackson ImmunoResearch, Cat# 001000-161) and 0.1% Triton in 1 × PBS and stained with the primary Iba1 antibody (1:1000 in blocking buffer containing 1% normal goat serum and 5% BSA in 1 × PBS) overnight at 4 °C. Following three washes in 1 × PBS, secondary antibody staining with goat anti-rabbit (1:1000 in blocking buffer) was performed for 1 h at room temperature. Sections were counterstained with 1 × TrueBlack according to the manufacturer’s instructions (Biotium, Fremont, CA), and nuclei were stained with Hoescht 33342 solution (Thermo Fisher Scientific). Slides were mounted with prolonged gold anti-fade mountant reagent (Thermo Fisher, P36934) and cover slips. Stained sections were examined using 20 × lenses of a Nikon fluorescence microscope, and images were acquired using a CCD digital camera. The density of Iba1^+^ cells was determined manually by counting the number of positive cells in 20 × images of 5 fields per animal using ImageJ Fiji software for biological image analysis^44,45^.

### Statistical methods

Methods used in experiments involving mice were performed in a manner consistent with ARRIVE guidelines (https://arriveguidelines.org). Statistical comparisons between groups were evaluated by the Mann–Whitney test using Graphpad Prism version 9.2.0. Significance was considered *P* < 0.05. *, **, ***, **** indicates *P* < 0.05, < 0.01, < 0.001, and < 0.0001, respectively.

### IACUC approval

All experiments were conducted following approval from the Animal Resources at Children’s Hospital, per regulations of the Institutional Animal Care and Use Committees (IACUC, Assurance number: A3303-01). All methods were carried out in accordance with relevant guidelines and regulations.

## Supplementary Information

Supplementary Information.
