# Supplementary material for: CCR2 promotes monocyte recruitment and intestinal inflammation in mice lacking the interleukin-10 receptor
Source: Sci Rep. 2022 Jan 10;12:452. doi: 10.1038/s41598-021-04098-7 (PMC8748948; doi:10.1038/s41598-021-04098-7)
Supplement: Supplementary file 1 — Supplementary Information. [file 41598_2021_4098_MOESM1_ESM.docx]

**Supplementary Information****.**

**CCR2 promotes monocyte recruitment and intestinal inflammation in mice lacking the interleukin-10 receptor**

Shorouk El Sayed^1,2^, Izabel Patik^1^, Naresh S. Redhu^1,3^, Jonathan N. Glickman^4^, Konstantinos Karagiannis^5^, El Sayed Y. El Naenaeey^2^, Gamal A. Elmowalid^2^, Ashraf M. Abd El Wahab^2^, Scott B. Snapper^1,6^, Bruce H. Horwitz^1,7*^


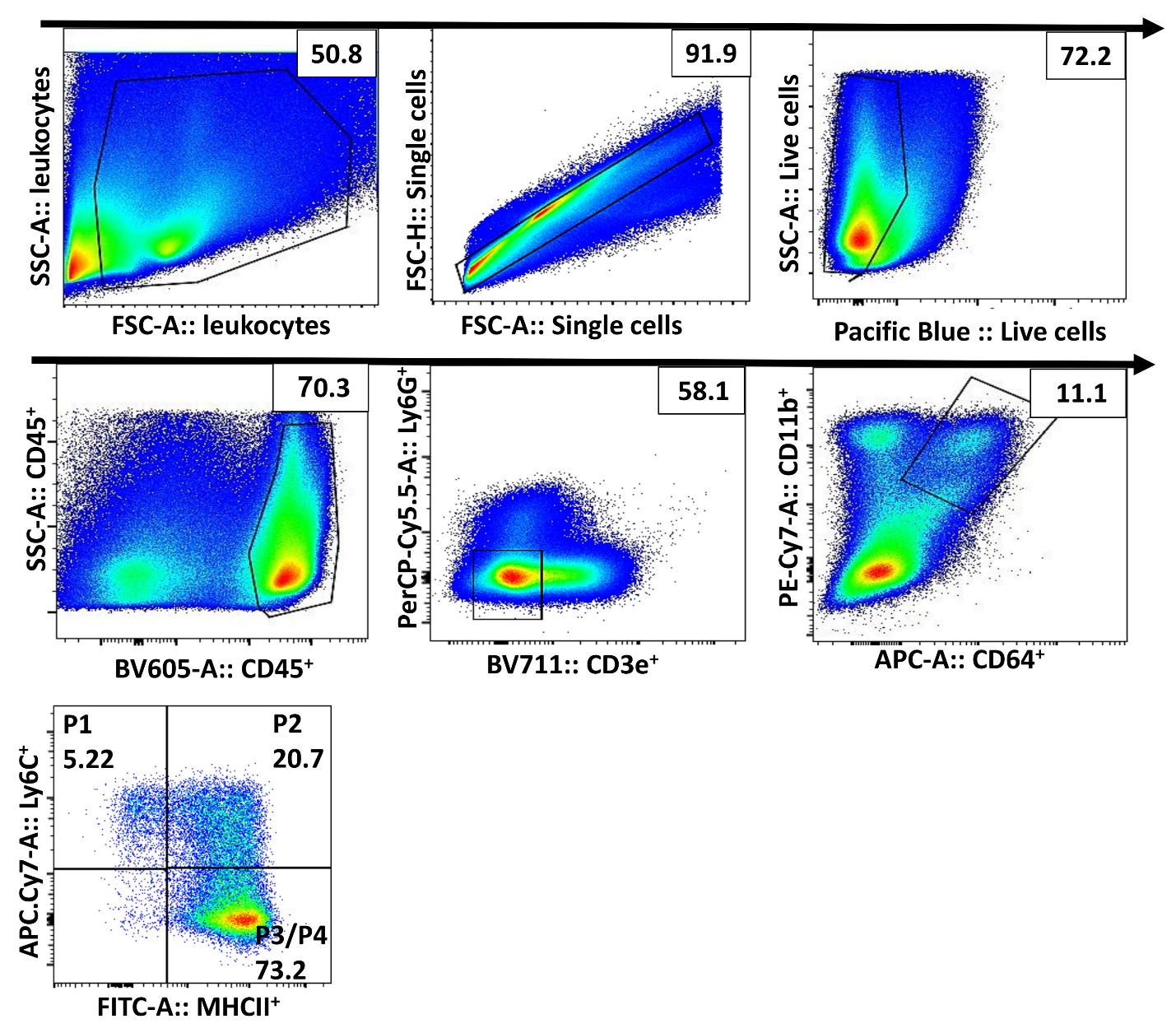


**Figure S1. Gating strategy to identify lamina propria macrophage subsets.**


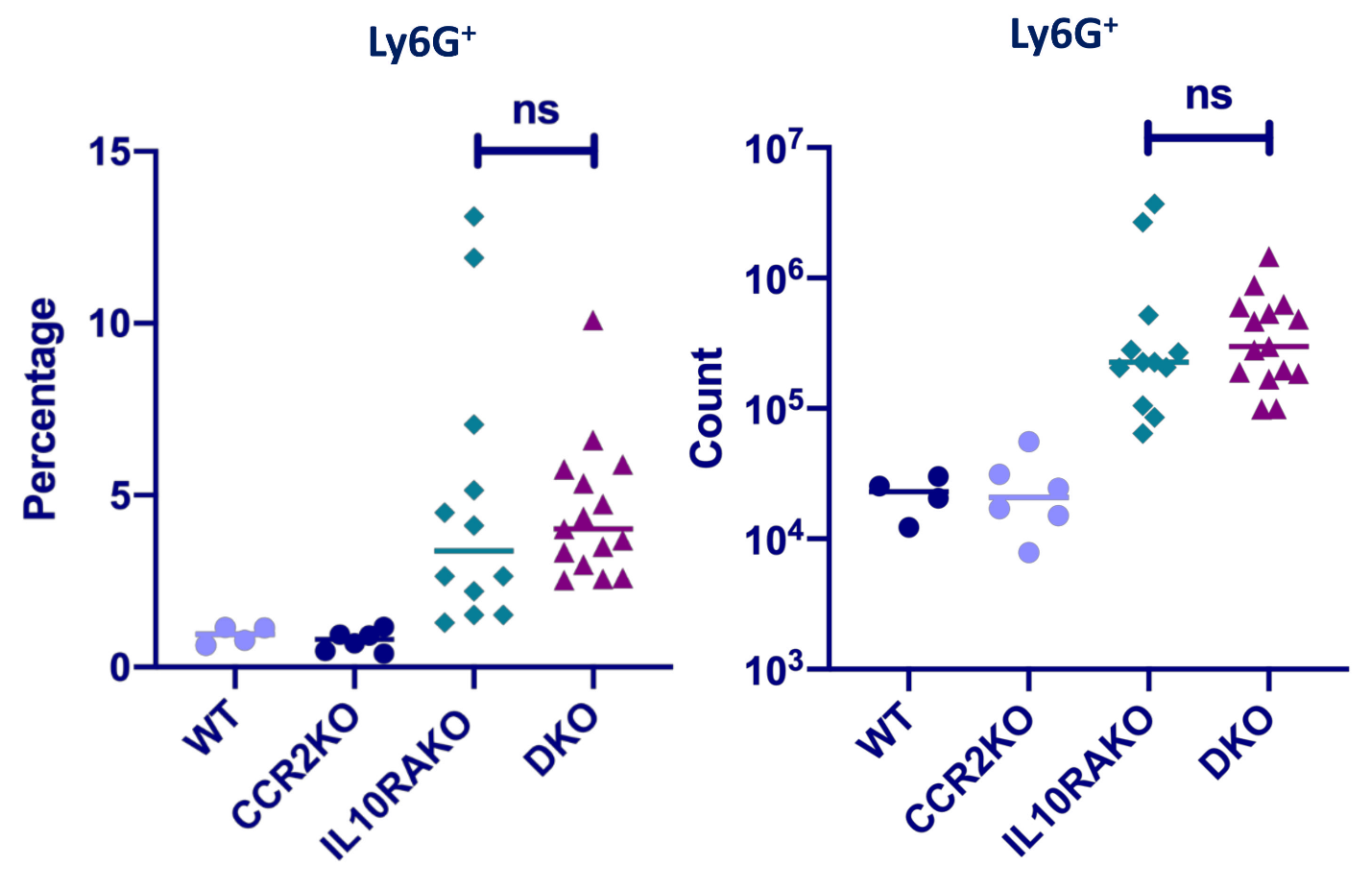


**Figure S2. Absence of CCR2 does not influence neutrophil infiltration of colon of mice lacking IL10RA.** The graph illustrated the percentage (left) and absolutes number (right) of colonic neutrophils out of total CD45^+^ cells in indicated genotypes. *Cdcs1^+/+^Il10ra^-/-^Ccr2^-/-^* (DKO/ n=15), *Cdcs1^+/+^Il10ra^-/-^* (IL10RAKO/ n=12), *Cdcs1^+/+^Ccr2^-/-^* (CCR2KO/ n=6), *Cdcs1^+/+^Il10ra^+/-^Ccr2^+/-^* (WT/ n=4). The results are pooled from 5 separate experiments. Please note the log scale in the graph on the right.


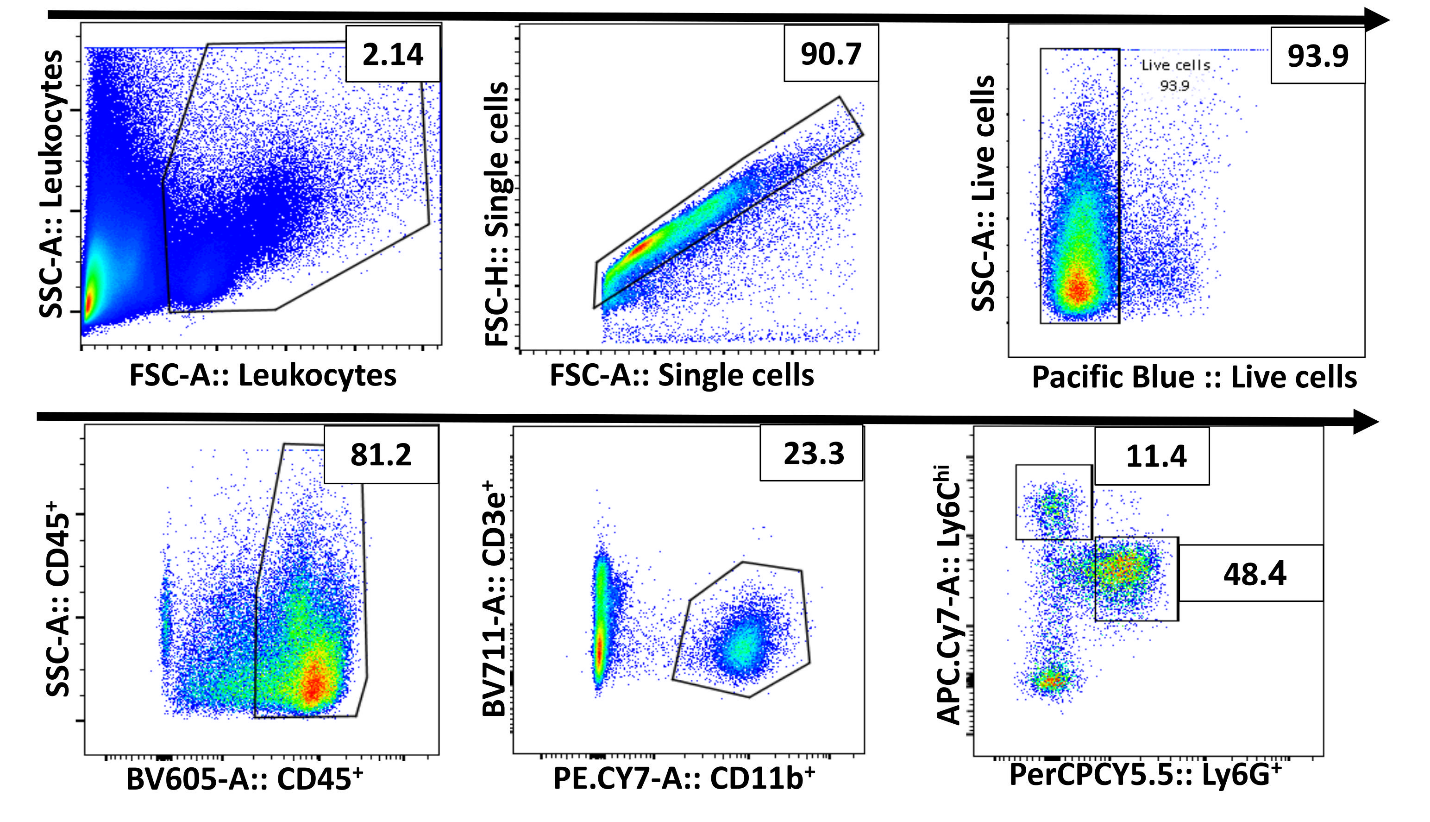


**Figure S3. Immune phenotyping of classical blood Ly6C^hi^ monocytes in inflammed mice.**
